# Antidiarrheal and Antibacterial Activities of Monterey Cypress Phytochemicals: In Vivo and In Vitro Approach

**DOI:** 10.3390/molecules27020346

**Published:** 2022-01-06

**Authors:** Elshaymaa I. Elmongy, Walaa A. Negm, Engy Elekhnawy, Thanaa A. El-Masry, Nashwah G. M. Attallah, Najla Altwaijry, Gaber El-Saber Batiha, Suzy A. El-Sherbeni

**Affiliations:** 1 Department of Pharmaceutical Sciences, College of Pharmacy, Princess Nourah bint Abdulrahman University, Riyadh 84428, Saudi Arabia; eielmongy@pnu.edu.sa (E.I.E.); ngmohamed@pnu.edu.sa (N.G.M.A.); naaltwaijry@pnu.edu.sa (N.A.); 2 Pharmaceutical Chemistry Department, Faculty of Pharmacy, Helwan University, Helwan 11795, Egypt; 3 Pharmacognosy Department, Faculty of Pharmacy, Tanta University, Tanta 31111, Egypt; 4 Pharmaceutical Microbiology Department, Faculty of Pharmacy, Tanta University, Tanta 31111, Egypt; 5 Pharmacology Department, Faculty of Pharmacy, Tanta University, Tanta 31111, Egypt; thanaa.elmasri@pharm.tanta.edu.eg; 6 Egyptian Drug Authority (EDA), Dokki, Giza 8655, Egypt; 7 Department of Pharmacology and Therapeutics, Faculty of Veterinary Medicine, Damanhour University, Damanhour 22511, Egypt; gaberbatiha@gmail.com

**Keywords:** *Cupressus* *macrocarpa*, ERIC-PCR, flow cytometry, LC-MS/MS, permeability, qRT-PCR

## Abstract

Monterey cypress (*Cupressus macrocarpa*) is a decorative plant; however, it possesses various pharmacological activities. Therefore, we explored the phytochemical profile of *C. macrocarpa* root methanol extract (CRME) for the first time. Moreover, we investigated its antidiarrheal (in vivo), antibacterial, and antibiofilm (in vitro) activities against *Salmonella enterica* clinical isolates. The LC-ESI-MS/MS analysis of CRME detected the presence of 39 compounds, besides isolation of 2,3,2″,3″-tetrahydro-4′-*O*-methyl amentoflavone, amentoflavone, and dihydrokaempferol-3-*O*-α-l-rhamnoside for the first time. Dihydrokaempferol-3-*O*-α-l-rhamnoside presented the highest antimicrobial activity and the range of values of MICs against *S. enterica* isolates was from 64 to 256 µg/mL. The antidiarrheal activity of CRME was investigated by induction of diarrhea using castor oil, and exhibited a significant reduction in diarrhea and defecation frequency at all doses, enteropooling (at 400 mg/kg), and gastrointestinal motility (at 200, 400 mg/kg) in mice. The antidiarrheal index of CRME increased in a dose-dependent manner. The effect of CRME on various membrane characters of *S. enterica* was studied after typing the isolates by ERIC-PCR. Its impact on efflux and its antibiofilm activity were inspected. The biofilm morphology was observed using light and scanning electron microscopes. The effect on efflux activity and biofilm formation was further elucidated using qRT-PCR. A significant increase in inner and outer membrane permeability and a significant decrease in integrity and depolarization (using flow cytometry) were detected with variable percentages. Furthermore, a significant reduction in efflux and biofilm formation was observed. Therefore, CRME could be a promising source for treatment of gastrointestinal tract diseases.

## 1. Introduction

Although antibiotics afford the key basis for the treatment of different bacterial infections, the emerging resistance to many antibiotics commonly used is a global concern. The repeated exposure and misuse of antibiotics have resulted in a high rate of antibacterial resistance and an increasing development of multidrug-resistant bacteria [1]. *Salmonella enterica* bacteria represent the main foodborne pathogens that are responsible for enteric infections and food poisoning [2]. In addition to the disseminated antibacterial resistance among *S. enterica* isolates, they can form biofilms and synthesize many cell surface components [3]. Biofilms are structured communities of one or more species of bacteria embedded in a self-produced polymeric matrix attached to either biotic or abiotic surfaces. It is observed that when bacteria are within biofilms, their eradication becomes difficult as biofilms give them good conservation against different antibiotics, disinfectants, and preservatives, in addition to protection against the host immune system and the stressful environmental conditions [4]. Therefore, there is a high need for new antimicrobials to fight against these bacteria. An underappreciated source for new antimicrobials might be the natural plants which contain many primary metabolites, secondary metabolites, and minerals responsible for antibacterial and antidiarrheal effects [5]. 

Infectious diseases cause major morbidity and mortality around the world. These diseases, such as diarrhea, influenza, and other infections, are particularly dangerous in developing countries. Diarrhea is the world’s second leading cause of death, killing hundreds of thousands of people every year [6,7]. Rapid antibiotic resistance is a particular issue in infectious diarrhea. Conventional treatments are usually employed by many communities, but they must be properly investigated to determine whether they are beneficial or not. Natural products may provide complementary pharmacological actions in alleviating diarrheal disease. For this reason, scientists have focused their efforts on the study of natural bioactive substances that could be used to treat diarrhea and other infectious disorders [8].

Medicinal plants are commonly used to treat gastrointestinal problems such as diarrhea and other infectious GIT disorders. The Monterey cypress (*Hesperocyparis macrocarpa*) is a common name for the *Cupressus macrocarpa* Hartw. ex Gordon plant [9]. *Cupressus* is a genus of evergreen conifers in the Cupressaceae family that goes by the common name cypress [9]. Despite being a decorative plant, *C. macrocarpa* has different pharmacological activities such as anti-inflammatory, hepatoprotective, nephroprotective, insecticidal, cytotoxic, and antiviral [10,11,12,13]. There have been numerous contributions to the composition of *C. macrocarpa* essential oil or other constituents in leaves [14,15,16,17], but to our knowledge, there has never been a comprehensive study on the roots of this plant. Here, we investigate the phytochemical profiling for methanol extract of *C. macrocarpa* roots by LC-ESI-MS/MS. In addition, the isolation of different compounds was performed for the first time. Moreover, the in vivo antidiarrheal activity, the antibacterial and antibiofilm effects of CRME, and isolated pure compounds against *S. enterica* clinical isolates were also evaluated.

## 2. Results

### 2.1. Metabolite Profiling of the C. macrocarpa Roots Methanol Extract

The hyphenated technique of liquid chromatography with tandem mass spectrometry (LC-MS/MS) was used to tentatively identify the components of the CRME. Untargeted metabolomics using liquid chromatography coupled to mass spectrometry were directed to collect information on as many metabolites as possible produced in this plant through taking advantage of information present in the data sets. The compounds of the CRME were tentatively identified by negative mode ESI-MS/MS. The total ion chromatogram of the metabolic profile is displayed in the Supplementary Figure S1. It showed the presence of 39 compounds that belong to various phytochemical subclasses such as organic acids, phenolics, coumarins, flavonoids, biflavonoids, polyflavonoids, catechins, flavonoid glycosides, stilbene, and triterpene saponins (Table 1). These compounds were identified based on the *m*/*z* of molecular ion [M–H]^−^ and interpretation of the MS and MS/MS spectra comparison with the library (in-house database) and previously reported literature [14,15,16,17,18,19]. Structures of the identified compounds are displayed in Figure S2.

#### 2.1.1. Flavones, Biflavones and Their Glycosides

Amentoflavone is 3′,8″-biapigenin, which showed a deprotonated molecular ion at *m*/*z* 537.189. Tetrahydro-4′-*O*-methyl amentoflavone is another derivative of amentoflavone with [M−H]^−^ ion at *m*/*z* 555.401. The [M−H]^−^ ion of apigenin aglycone was observed at *m*/*z* 269.157. The [M−H]^−^ ion of acacetin and luteolin was detected at *m*/*z* 283.162 and 285.183, respectively. Both luteolin-7-*O*-glucoside and apigenin-7-*O*-glucoside were detected by their [M−H]- ion at *m*/*z* 447.084 and 431.103, respectively. The loss of glucose moiety was noticed by the neutral loss of 162 Da at *m*/*z* 285.035 and 269.042, respectively. The other glycosides, acacetin-7-*O*-rutinoside, baicalein-7-*O*-glucuronide and apigenin-7-*O*-neohesperodoside or rhoifolin showed the [M−H]¯ ion at *m*/*z* 591.144, 445.109, 577.118, respectively. The fragment ions of rutinose and neohesperidose were noticed by loss of 308 Da.

#### 2.1.2. Flavonols and Their Glycosides

The [M−H]^−^ ions of quercetin, quercetin-3-d-xyloside, dihydrokaempferol-3-*O*-α-l-rhamnoside, kaempferol-3-glucuronide, 3,5,7-trihydroxy-4′-methoxyflavone or (diosmetin), kaempferol-7-neohesperidoside were detected at *m*/*z* 301.031, 433.164, 434.189, 461.103, 299.199, 593.131. The neutral loss of xylose (132 Da), rhamnose (146 Da), glucuronic acid (176 Da) and neohesperidose (308 Da) was noticed for different glycosides. The deprotonated molecular ions of isorhamnetin, isorhamnetin-3-*O*-rutinoside or narcissin, isorhamnetin-3-*O*-glucoside were observed at *m*/*z* 315.195, 623.192 and 477.109, respectively.

#### 2.1.3. Flavanones and Their Glycosides

The deprotonated molecular ions of naringenin, naringenin-7-*O*-glucoside or prunin and hesperetin were detected at *m*/*z* 271.057, 433. 196 and 301.178, respectively. The fragment ion at *m*/*z* 271.065 confirmed the neutral loss of glucose moiety of naringenin-7-*O*-glucoside.

#### 2.1.4. Procyanidins

Table 1 showed the existence of procyanidin B1, B2 and C1. Their deprotonated molecular ion was noticed at *m*/*z* 577.125, 577.134 and 865.194, respectively. Their fragmentation pattern showed a common fragment at *m*/*z* 289 for catechin and epicatechin. These procyanidins are either dimer in case of procyanidin B1 and B2 or trimer of procyanidin C1. Epicatechin was also recognized with molecular ion at [M−H]^−^ = 289.072. Fragment at *m*/*z* 245.080 indicated the neutral loss of CO_2_.

#### 2.1.5. Stilbenes and Other Compounds

Stilbenes as E-3,4,5′-trihydroxy-3′ glucopyranosylstilbene or astringin showed [M−H]^−^ at *m*/*z* 405.124 with loss of 162 Da of glucose. Coumarins as esculin had [M−H]^−^ at *m*/*z* 339.191.

### 2.2. Spectroscopic Data of Isolated Compounds

**Figure 1:**
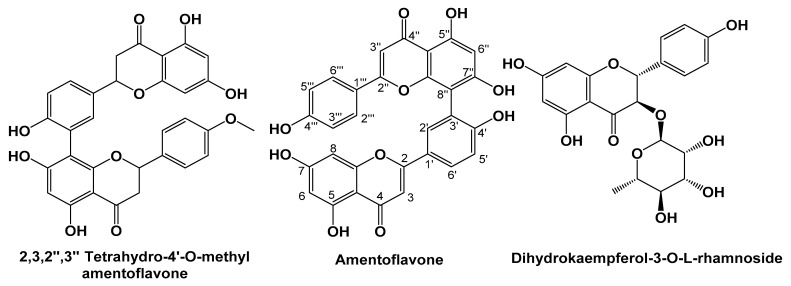
Chemical structures of the isolated compounds from CRME.

For the first time from CRME, three compounds 2,3,2″,3″ tetrahydro-4′-*O*-methyl amentoflavone, amentoflavone, and dihydrokaempferol-3-*O*-α-l-rhamnoside were separated. The chemical structures of the isolated compounds are presented in Figure 1.

#### 2.2.1. Characterization of 2,3,2″,3″-tetrahydro-4′-*O*-methyl Amentoflavone

^1^H-NMR [CD_3_OD, 400 MHz] δ_H_: 5.36–5.42 (H-2; 1H, m), 3.03–3.12 (H-3*ax*; 1H, m), 2.64–2.72 (H-3*eq*; 1H, m), 5.81 (H-6; 1H, m), 6.27 (H-8; 1H, d, *J* = 2.0 Hz), 7.36–7.51 (H-2′; 1H, m), 7.10 (H-5′; 1H, dd, *J* = 8.5, 4.0 Hz), 7.36–7.51 (H-6′; 1H, m), 5.36–5.41 (H-2″; 1H, m), 3.03–3.12 (H-3″*ax*; 1H, m), 2.64–2.72 (H-3″*eq*; 1H, m), 6.52 (H-6″; 1H, d, *J* = 4.5 Hz), 6.67 (H-3‴, 5‴; 2H, m), 7.36 (H-2‴, 6‴; 2H, dd, *J* = 8.5, 3.0 Hz), 3.66 (OCH_3_ at C-4ʹ; 3H, d, *J* = 13.5 Hz).

^13^C-NMR [DMSO-*d_6_*, 100 MHz] δ_C_: 78.7, 78.5 (C-2), 42.5, 41.8 (C-3), 195.7 (C-4), 164.4 (C5), 95.7, 94.8 (C-6), 166.8 (C-7), 94.9, 95.0 (C-8), 163.5 (C-9), 101.7, 101.8 (C-10), 130.7, 130.7 (C-1′), 131.2, 131.3 (C-2ʹ), 121.8 (C-3′), 158.1, 158.3 (C-4′), 110.7, 110.7 (C-5′), 127.9, 127.8 (C-6ʹ), 79.1 (C-2″), 42.6, 43.0 (C-3″), 196.9 (C-4″), 163.5 (C-5″), 98.3, 98.3 (C-6″), 164.1, 164.0 (C-7″), 105.8, 105.9 (C-8″), 160.8 (C-9″), 102.6, 102.5 (C-10″), 129.6, 130.0 (C-1‴), 127.6, 128.0 (C-2‴, 6‴), 115.1, 115.2 (C-3‴, 5‴), 157.9, 157.7 (C-4‴), 55.1, 55.0 (OCH_3_ at C-4′); ESI-MS *m*/*z* 555. 223 [M−H]^−^.

#### 2.2.2. Characterization of Amentoflavone

^1^H-NMR [DMSO-*d_6_*, 400 MHz] δ_H_: 6.8 (H-3; 1H, s), 6.18 (H-6; 1H, d, J = 1.8 Hz), 6.47 (H-8; 1H, d, *J* = 1.8 Hz), 8.00 (H-2′; 1H, d, *J* = 1.5 Hz), 7.15 (H-5′; 1H, d, *J* = 8.5 Hz), 7.99 (H-6′; 1H, dd, *J* = 8.5, 1.5 Hz), 6.79 (H-3″; 1H, s), 6.40 (H-6″; 1H, s), 6.73 (H-3‴, 5‴; 2H, d, *J* = 8.0 Hz), 7.58 (H-2‴ 6‴; 2H, d, *J* = 8.0 Hz), 10.86, 10.31, 10.65, 10.66, 12.97, 13.1 (OH at C-7, C-7″, C-4′, C-4‴, C-5, and C-5″, respectively; each 1H, s.

^13^C-NMR [DMSO-*d_6_*, 100 MHz] δ_C_: 163.3 (C-2), 102.8 (C-3), 181.7 (C-4), 161.4 (C-5), 98.8 (C-6), 163.5 (C-7), 94.2 (C-8), 157.8 (C-9), 103.3 (C-10), 120.5 (C-1′), 127.6 (C-2ʹ), 121.4 (C-3’), 159.9 (C4′), 116.7 (C-5′), 131.4 (C-6′), 164.1 (C-2″), 102.6 (C-3″), 182. 0 (C-4″), 160.5 (C-5″), 99.2 (C-6″), 162.1 (C-7″), 104.5 (C-8″), 154.5 (C-9″), 103.7 (C-10″), 120.6 (C-1‴), 128.6 (C-2‴), 115.7 (C-3‴), 160.9 (C-4‴), 115.7 (C-5‴), 128.6 (C-6‴); ESI-MS *m*/*z* 537. 193 [M−H]^−^.

#### 2.2.3. Characterization of Dihydrokaempferol-3-*O*-α-l-rhamnoside

^1^H-NMR [CD_3_OD, 400 MHz] δ_H_: 4.96 (H-2; 1H, d, *J* = 11.0 Hz), 4.54 (H-3; 1H, d, *J* = 11.0 Hz), 5. 85 (H-6; 1H, d, *J* = 2.0 Hz), 5.9 (H-8; 1H, d, *J* = 2.0 Hz), 7.35 (H-2′, 6′; 2H, d, *J* = 9.0 Hz), 6.83 (H-3′, 5′; 2H, d, *J* = 9.0 Hz), Rhamnose moiety: 4.59 (H-1″; 1H, s), 3.87 (H-2″; 1H, m), 3.96 (H-3″; 1H, m), 3.68 (H-4″; 1H, m), 4.21 (H-5″; 1H, m), 1.29 (C-5- CH_3_; 3H, d, *J* = 6.5).

^13^C-NMR [CD_3_OD, 100 MHz] δ: 85.6 (C-2), 78.4 (C-3), 193.4 (C-4), 165.1 (C-5), 98.2 (C-6), 170.2 (C-7), 97.2 (C-8), 160.2 (C-9), 102.3 (C-10), 130.6 (C-1′), 131.3 (C-2′), 116.8 (C-3′), 158.1 (C-4′), 116.8 (C-5′), 131.3 (C-6′). Rhamnose moiety (C-1″-6″): 103.8, 70.5, 71.8, 74.4, 69.8, 18.1. ESI-MS *m*/*z* 433.0607 [M−H]^−^.

### 2.3. In Vitro Antibacterial Effect of CRME against S. enterica Isolates

#### 2.3.1. Genotypic Patterns of *S. enterica* Isolates Using the ERIC-PCR Technique

**Figure 2:**
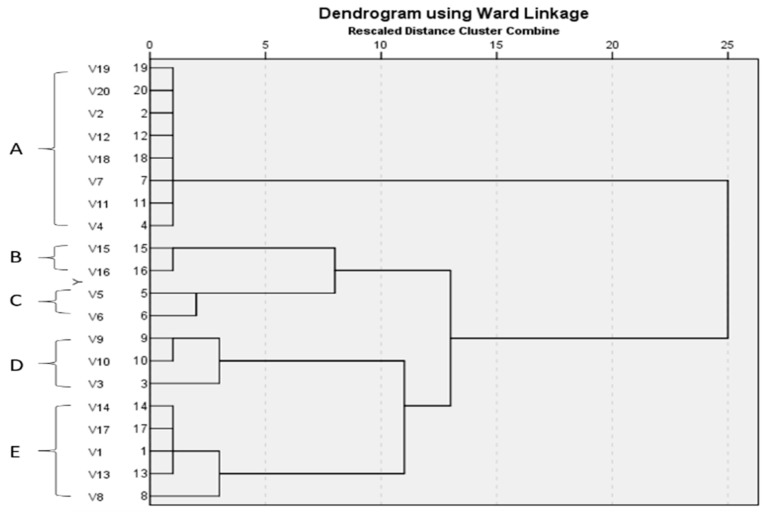
Dendrogram showing the degree of relatedness of *S. enterica* isolates as determined by ERIC-PCR fingerprinting.

The ERIC-PCR technique was utilized for the molecular typing of 20 *S. enterica* isolates. The apparent molecular sizes of the bands ranged from 200 to 1200 bp. The ERIC dendrogram revealed five distinct clusters (A, B, C, D and E) with genetic similarities ranging from 33% to 100% (Figure 2).

#### 2.3.2. Antimicrobial Activity of *C. macrocarpa* Roots

Using the agar well diffusion method, CRME showed an antibacterial effect on the tested *S. enterica* isolates. The minimum inhibitory concentration (MIC) values of CRME were determined by the broth microdilution method, which ranged from 64 to 1024 µg/mL, as shown in Table S1.

#### 2.3.3. Antimicrobial Activity of Isolated Pure Compounds

Dihydrokaempferol-3-*O*-α-l-rhamnoside presented the highest antimicrobial activity and the range of values of MICs against *S. enterica* isolates was from 64 to 256 µg/mL, while the MIC values range of amentoflavone was from 128 to 1024 µg/mL. 2,3,2″,3″ Tetrahydro-4′-*O*-methyl amentoflavone exhibited the least antimicrobial activity, with MIC values of 512–1024 µg/mL.

#### 2.3.4. Integrity of Cell Membrane

**Figure 3:**
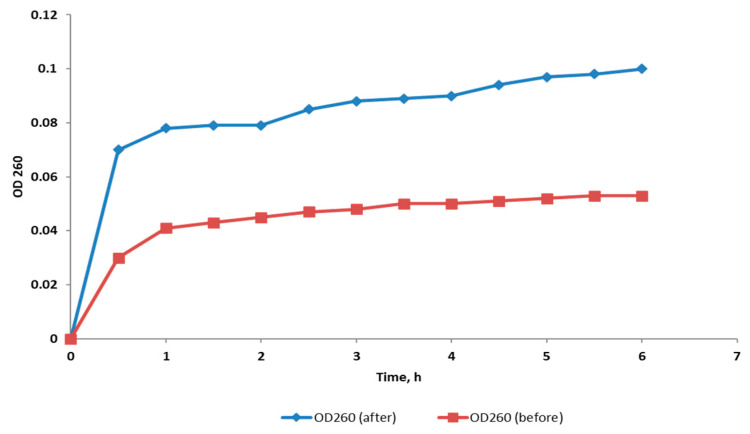
A chart showing the increase in the release of the material absorbing at 260 nm (indicating a decrease in the membrane integrity) from a representative *S. enterica* isolate after treatment with CRME (32 µg/mL).

The cell membrane integrity of *S. enterica* isolates was evaluated after treatment with CRME (at concentrations of 32 to 512 µg/mL) through tracking the release of the materials, which absorbed at 260 nm (DNA and RNA), from the bacterial cytoplasm. In 45% of the treated isolates, the bacterial membrane integrity was found to be significantly reduced (*p* < 0.05). Figure 3 depicts a representative example.

#### 2.3.5. Inner Membrane Permeability

**Figure 4:**
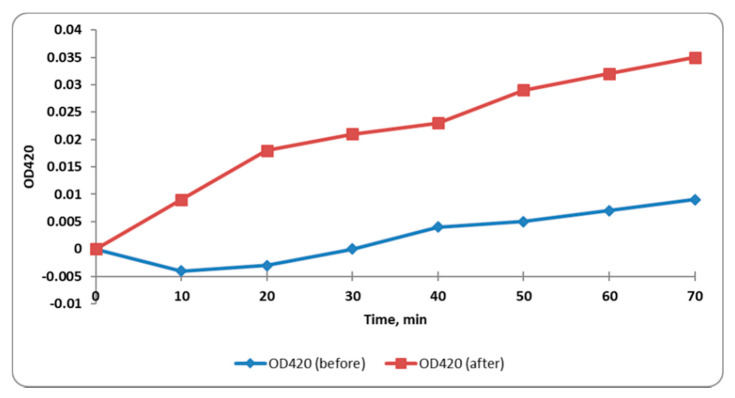
A chart showing the increase in the inner membrane permeability of a representative *S. enterica* isolate after treatment with CRME was determined by measuring the ONP absorbance with time.

When the permeability of the inner membrane of bacteria increased, the entry of *O*-nitrophenyl-β-galactopyranoside (ONPG) to the cytoplasm of the bacterial cells increased where it was broken down by the enzyme β-galactosidase to ONP, which has a yellow color. The inner membrane permeability was monitored by detection of the absorbance at OD_420_ (*O*-nitrophenol, ONP, absorbance) with time. We observed that the inner membrane permeability increased significantly (*p* < 0.05) in 50% of *S. enterica* isolates after treatment with CRME (at concentrations of 32 to 512 µg/mL). A representative example is shown in Figure 4.

#### 2.3.6. Outer Membrane Permeability

**Figure 5:**
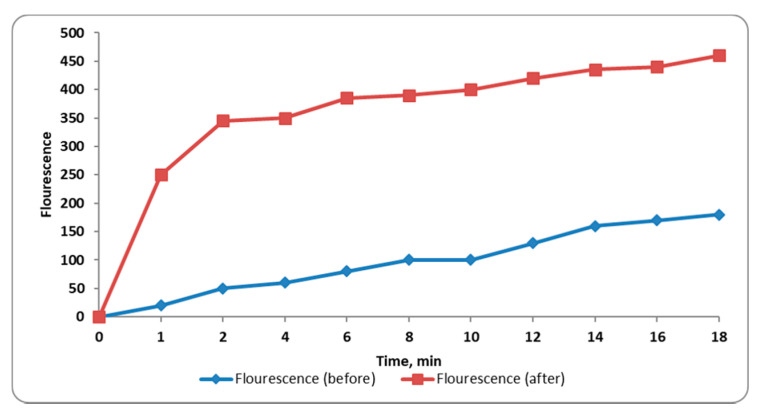
A chart showing the increase in the outer membrane permeability of a representative *S. enterica* isolate after treatment with CRME was detected by determining the fluorescence of NPN against time.

Outer membrane permeability was monitored by measuring the fluorescence of N-phenyl naphthylamine (NPN). We observed a significant increase (*p* < 0.05) in NPN fluorescence, indicating in turn a significant increase in outer membrane permeability, in 45% of the tested isolates after treatment with CRME (at concentrations of 32 to 512 µg/mL). A representative example is presented in Figure 5.

#### 2.3.7. Membrane Depolarization

**Figure 6:**
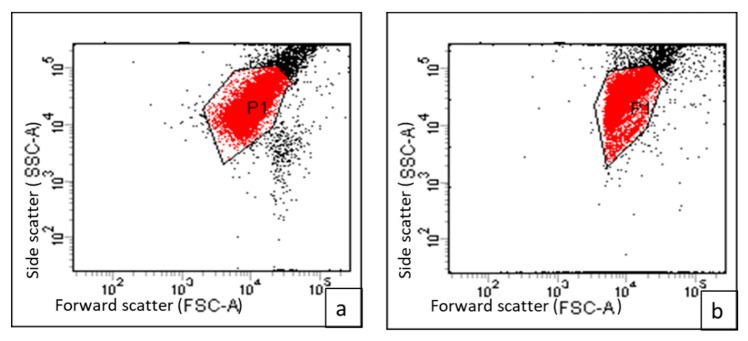
A representative flow cytometric chart (dot plot) showing the fluorescent gap (**a**) before (95.7%) and (**b**) after (22.8%) treatment with CRME measured by FACS verse flow cytometer.

Flow cytometric measurements were carried out using DiBAC4(3), which is a membrane potential-sensitive fluorescent stain. This compound was able to enter the cytoplasm of the depolarized cells where it could bind to the intracellular proteins, increasing the fluorescence. We observed a significant decrease (*p* < 0.05) in the membrane depolarization of 40% of the tested isolates after treatment with CRME (at concentrations of 32 to 512 µg/mL). An illustrative example of the decrease in membrane depolarization after treatment is shown in Figure 6.

#### 2.3.8. Effect on Efflux Activity

In this study, *S. enterica* isolates exhibited a substantial decrease in efflux activity (*p* < 0.05), using the fluorometric cartwheel method, after treatment with CRME (at concentrations of 32 to 512 µg/mL) as presented in Table 2.

#### 2.3.9. Antibiofilm Activity of *C. macrocarpa* Roots Methanol Extract

**Figure 7:**
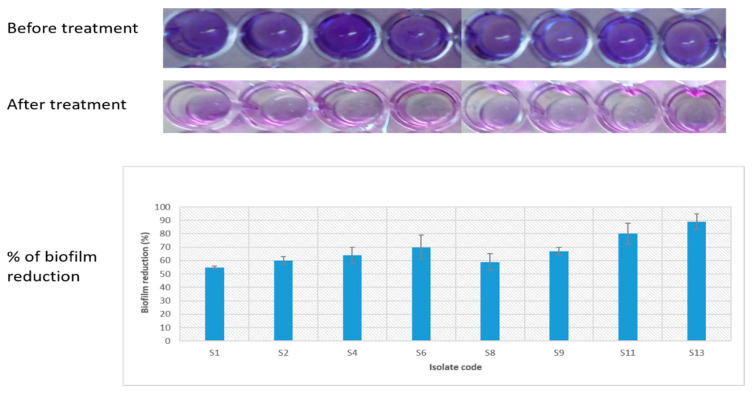
Crystal violet assay for evaluation of the antibiofilm efficiency of CRME against *S. enterica* isolates showing a significant reduction (*p* < 0.05) in biofilm formation in 8 isolates (S1, S2, S4, S6, S8, S9, S11, and S13).

Crystal violet assay was utilized for evaluation of the antibiofilm efficiency of CRME (at concentrations of 32 to 512 µg/mL) against *S. enterica* isolates. We observed a significant reduction (*p* < 0.05) in biofilm formation in 8 (40%) isolates, as shown in Figure 7.

#### 2.3.10. Effect on the Biofilm Morphology

**Figure 8:**
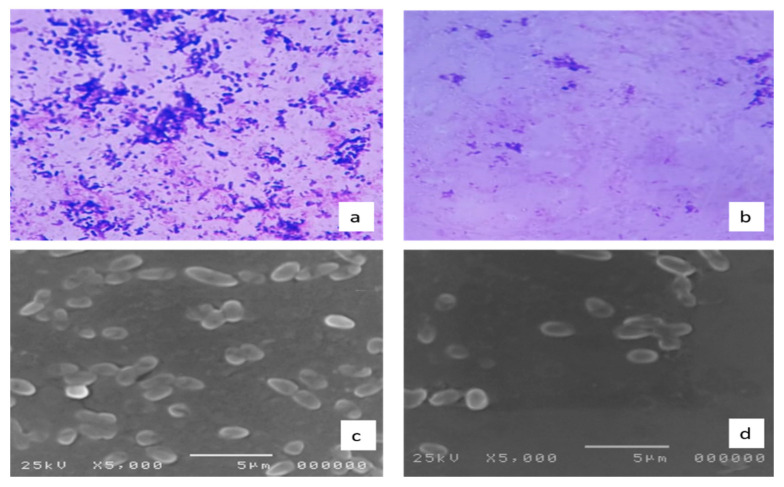
A representative example of the decrease in biofilm formation by *S. enterica* isolates (**a**,**b**) viewed by the light microscope, and (**c**,**d**) viewed by the scanning electron microscope before (**a**,**c**) and after (**b**,**d**) treatment with CRME.

Examination of the morphology of the biofilms formed by *S. enterica* isolates (*n* = 8), which showed an inhibition of the biofilm formation by crystal violet assay, was carried out by light and scanning electron microscopes (SEM). Visible reductions in the formed biofilms were found, as shown in Figure 8.

#### 2.3.11. Quantitative Real-Time PCR (qRT-PCR)

qRT-PCR was used to explore the impact of CRME (at concentrations of 32 to 512 µg/mL) on the efflux activity of *S. enterica* isolates (*n* = 11), which showed a reduction in the efflux activity by fluorometric cartwheel method, as shown in Table 3. In addition, the expression of the genes associated with the biofilm formation was investigated in *S. enterica* isolates (*n* = 8), which showed a reduction in the biofilm formation by crystal violet assay, as shown in Table 4. The gene expression was inspected in the tested isolates relative to the expression of the 16S rRNA gene.

### 2.4. Antidiarrheal Effect of Cupressus Macrocarpa Roots Methanol Extract

#### 2.4.1. Test of Acute Oral Toxicity

The CRME did not produce any toxicity or changes in the behavior of the tested animals at the range of doses of 50 to 3000 mg/kg during this test. Furthermore, we did not observe any mortality or changes in the physical conditions of the animals, such as loss of weight, for 72 h after administration of the extract. A finding suggests that the CRME has no toxicity up to 3000 mg/kg.

#### 2.4.2. Castor Oil-Induced Diarrhea

The results of the probable antidiarrheal activity of CRME are shown in Table 5.

#### 2.4.3. Impact on Castor Oil-Induced Enteropooling

The CRME resulted in a significant reduction in the mean volume and weight of the small intestinal contents (MVSIC and MWSIC) at the dose of 400 mg/kg body weight (b.w.) compared to the control. The remaining dose did not exhibit a significant reduction, as shown in Table 6.

#### 2.4.4. Gastrointestinal Motility Test

The CRME exhibited an inhibitory effect on the intestinal transit of charcoal in a dose-dependent manner, as shown in Table 7.

#### 2.4.5. Antidiarrheal Index

The in vivo antidiarrheal index of CRME increased in a dose-dependent manner, as shown in Table 8.

## 3. Discussion

From the total methanolic extract of *C. macrocarpa* roots, three compounds were separated for the first time. The ^1^H-NMR spectrum of the first compound suggested the biflavanone structure via signals at δ 2.64–2.72 (H-3*eq*, H-3″*eq*, m), 3.03–3.12 (H-3ax, H-3″ax, m) and 5.36–5.40 (m, H-2, H-2″). An ABX system was displayed by signals at δ 7.36–7.5 (1H, m) for H-2′, 7.36–7.5 (IH, m) for H-6′ and 7.1 (1H, dd, *J* = 8.5, 4.0 Hz) for H-5′, as well as one AA’BB’ patterns as indicated by the signals at δ 7.36 (2H, dd, *J =* 8.5, 3.0 Hz), for H-2‴, 6‴ and δ 6.67 (2H, m) for H-3‴, 5‴. ^1^H-NMR spectrum revealed a tetrahydro-amentoflavone pattern with one aromatic methoxy signal at δ 3.66, suggesting mono-methoxy derivatives. Examination of the ^13^C-NMR spectra further confirmed the biflavanone structure from the presence of 30 carbons in addition to one methoxy signal at δ 55.03. It also showed two duplicated benzylic oxymethine signals at δ 78.73, 78.53 (C-2), and 79.1 (C-2″); two duplicated methylene carbon at δ 42.5, 41.8 (C-3) and 42.6,43.01 (C-3″); two carbonyls at δ 195.7 (C-4) and 196.9 (C-4″). This compound’s ESI-MS spectrum revealed ions at *m*/*z* 555.223 [M−H]^−^ which matches with the determined structure. Therefore, this compound was identified as 2,3,2″,3″ tetrahydro-4’-*O*-methyl amentoflavone [18].

The presence of six hydroxyl groups was determined by ^1^H-NMR for the second compound, two of which resonated at δ 12.97, 13.1, showing the presence of two chelated hydroxyl groups at 5, 5′ positions, respectively. The remaining non-chelated hydroxyl groups were observed at δ 10.86, 10.31, 10.65 and 10.66 attributed to 7,7″, 4′ and 4‴ OH, respectively. The ^1^H-NMR spectrum also showed an AA’BB’ system for ring E protons, as indicated by δ 7.58 (2H, d, *J =* 8.0 Hz) for H-2‴, 6‴ and δ 6.73 (2H, d, *J =* 8.0 Hz) for H-3‴, 5‴. Additionally, an ABX system was also shown by the signals at δ 8.0 (d, *J =* 1.5 Hz) for H-2′, 7.15 (d, *J =* 8.5 Hz) for H-5′ and 7.99 (dd, *J =* 8.5, 1.5 Hz) for H-6’ in ring B protons. The appearance of two *meta*-coupled doublets (*J* = 1.8 Hz) each due to one proton in the upfield aromatic region at δ 6.18 and 6.47 were assigned to C-6 and 8 protons of ring A, respectively. Meanwhile, the only signal at δ 6.4 was assigned to H 8″ or H 6″ implying that either C-6″ or C-8″ of ring D had to be involved in the inter flavonoid linkage. The ^13^C-NMR spectrum confirmed that the biflavonoid nature of the compound showed 30 carbons and established that C-8″, C-3′ were involved in interflavonoid linkage due to the downfield shift of C-8″ by 10 ppm and of C-3’ by 6 ppm, respectively. ESI-MS presented *m*/*z* 537. 193 [M−H]^−^ which is consistent with an amentoflavone structure [19].

The ^1^H-NMR spectrum of the third compound established the flavanone’s nature, which showed doublet signals (1H, d, *J* = 11.0) resonating at δ 4.96, 4.54 corresponding to protons at C-2, C-3. The presence of *meta*-coupled doublets (1H, d, *J* = 2.0 Hz), each due to one proton, in the upfield aromatic region at δ 5.85 and 5.90 was due to the C-6 and C-8 protons of ring A. The two protons resonating at δ 7.35 (2H, d, *J* = 9.0 Hz) can be assigned as ring B protons at 2′, 6′ positions coupled to the signal at δ 6.83 due to the two protons at C-3′ and C-5′. In addition, the presence of sugar protons resonating at δ 3.68–4.21 (m) and one anomeric singlet proton at δ 4.59 indicated the presence of *α*-l-rhamnose. The ^13^C-NMR spectrum showed 21 carbons established the flavanone glycoside nature as signals resonating at δ 85.6, 78.4 which can be ascribed to C-2, C-3, respectively. ESI-MS showed a pseudo-molecular ion at 433. 0607 for [M−H]^−^ which matches with the determined structure. Therefore, this compound was established to be 2,3-dihydrokaempferol-3-*O*-α-l-rhamnoside [20].

LC-MS/MS of CRME revealed 39 compounds belonging to different phytochemical subclasses. Our results were consistent with previously reported data about the antimicrobial effect of different flavonoids or flavonoid glycosides as apigenin [21], quercetin [22,23], luteolin [24,25], isorhamnetin [26], naringenin [27], kaempferol [28], neohesperidin [29], procyanidin [30], amentoflavone [20] and coumarin [22] compounds. It was also in agreement with the antidiarrheal effects of procyanidins [31], coumarins [32] and triterpenes [33].

In addition, the separated compounds were tested against *S. enterica* clinical isolates, and it was found that dihydrokaempferol-3-*O*-α-l-rhamnoside exerted the best antimicrobial activity with MICs ranging from 64 µg/mL to 256 followed by amentoflavone (128 to 1024 µg/mL).

Foodborne diseases are an emerging public health issue worldwide. *S. enterica* is among the different bacteria involved in these types of diseases, which represent a major cause of hospitalization and death. In this study, we used ERIC-PCR to determine the genetic relatedness of 20 *S. enterica* isolates. The generated dendrogram from the *S. enterica* ERIC genotypes presented five distinct clusters (A, B, C, D and E).

The spread of antibiotic resistance among *S. enterica* bacteria, coupled with its high ability to form biofilms that increase its capacity to resist antibiotics, has resulted in many calls for novel approaches to resolve this matter. Plant extracts could provide new approaches to combat and control such pathogenic bacteria [34]. To our knowledge, this study is the first investigation of the antibacterial and antibiofilm activity of CRME against *S. enterica* clinical isolates.

In the present study, CRME exhibited antibacterial activity against *S. enterica* clinical isolates with MIC values that ranged from 64 to 1024 µg/mL. To comprehend the antibacterial mechanism of CRME, we studied its effect on different bacterial membrane properties in addition to its effect on efflux activity. The cell membrane of bacteria is an important target for many antibacterial compounds [35]. The increase in the release of the intracellular materials which absorb at 260 nm from the bacterial cells, such as DNA and RNA, can be used as evidence for the occurrence of damage in the membrane [36]. We observed that the bacterial membrane integrity significantly decreased (*p* < 0.05) in 45% of the *S. enterica* isolates after treatment. Gram-negative bacteria such as *S. enterica* have an inner membrane and an additional outer membrane [37]. We tested the effect of CRME on the inner and outer membrane permeability. The inner and outer membrane permeability increased significantly (*p* < 0.05) with percentages of 50% and 45%, respectively. The increase in permeability could adversely affect the cell metabolism and might result in bacterial death [38]. The membrane depolarization is an important membrane character that was inspected using flow cytometry. The membrane depolarization significantly decreased (*p* < 0.05) in 40% of the tested isolates after treatment with CRME. Dissipation of the membrane potential could contribute to the potency of the antimicrobial compounds [39].

The effect on the efflux pump activity of *S. enterica* isolates was studied using the EtBr cartwheel method as EtBr acted as a substrate for the efflux pumps [40]. Remarkably, 11 *S. enterica* isolates (i.e., 55% of the isolates) changed from having positive efflux activity to intermediate or negative efflux activity after treatment with CRME. This inhibition of the efflux pump activity could have many benefits as it could regain susceptibility of the resistant bacteria to different antibiotics [41].

Bacterial cells in biofilm show higher resistance to antibiotics and usually form persistent infections, particularly in hospitals and health care settings [42]. Herein, CRME caused a significant reduction (*p* < 0.05) in biofilm formation in eight isolates (40% of isolates). Avisible reductions in the biofilm formation were observed. Many researchers have reported the inhibitory effects of different plants on biofilm formation by different bacteria [43,44,45,46,47]. The study of the expression levels of the genes related to the efflux activity in *S. enterica* isolates. A significant reduction in the expression of *acr*A, *acr*B genes in 35% and 40% of the tested isolates, respectively, in addition to a non-significant change in the expression of *tol*C, and *oqx*B genes, were observed. The expression of *agf*A and *spi*A genes was significantly reduced in 30% of *S. enterica* isolates after treatment with CRME.

Diarrhea generally occurs when a fluid imbalance is present in the gastrointestinal tract. It also occurs when there a disturbance in the motility of the smooth bowel muscles [48]. Many people use various plant parts for the treatment of different diseases such as diarrhea without any scientific base about either their efficacy or safety [49]. Herein, we investigated the antidiarrheal effect of CRME. Castor oil is a natural agent that is commonly used as a diarrheal stimulator by releasing its active metabolite, ricinoleic acid. Ricinoleic acid is an irritant laxative, as it enhances the peristaltic activity of the upper part of the small intestine, resulting in alterations in the electrolyte permeability of the intestinal mucosa. Moreover, it induces a localized irritation in the intestinal mucosa, thus increasing gastrointestinal motility [7]. The castor oil-induced diarrheal model was used to measure the antidiarrheal activity of CRME, at doses of 100, 200 and 400 mg/kg body weight, compared with loperamide (3 mg/kg b. w.) as a standard drug. The extract showed a remarkable antidiarrheal activity, evidenced by the reduction in the rate of defecation where the percentage of inhibition was found to be 85.9%, 88% and 93.5% at the dose of 100, 200 and 400 mg/kg b. w., respectively. The defecation inhibition percentage at dose 400 mg/kg was comparable with that of loperamide (91.5%). Moreover, the CRME was tested for the inhibition of gastrointestinal motility by evaluating its impact on the intestinal transit of charcoal in mice. The CRME resulted in a remarkable decrease in intestinal transit (64.95% and 80.24%) at 100 and 200 mg/kg, compared with 74.63% for loperamide. The CRME manifested a significant inhibition of castor oil-induced enteropooling at 400 mg/kg b. w., in terms of volume and weight of intestinal content, comparable to the control group.

The antidiarrheal index is a value calculated for the expectation of the overall purging effect of the extract [50]. The antidiarrheal index of CRME increased in a dose-dependent manner.

## 4. Materials and Methods

### 4.1. General

Material for column chromatography (CC) as silica gel, Sephadex LH-20 and pre-coated TLC sheets silica gel F_254_ were purchased from Merck, 70–230 mesh, Sigma-Aldrich Chemical Co. and Merck, respectively. Camag UV lamps with wavelengths of 254 and 366 nm were used for observations. AlCl_3_ or 10% sulfuric acid spray reagents were separately used for the detection of spots. A Bruker High-Performance Digital FT-Avance III NMR spectrometer was used to record NMR spectra. The ^1^H and ^13^C-NMR samples were examined at 400 MHz and 100 MHz, respectively. DMSO-d_6_ or CD_3_OD were used to dissolve the samples.

### 4.2. Plant Material

A nursery in Shebin El-Kom City provided the *C. macrocarpa* roots (the age of the plant was seven years old). In May of 2017, the plant was gathered and was identified by Prof. Mohammed I. Fotoh, Ornamental Horticulture and Landscape Design Professor. The voucher specimen (PG00411-R) was placed in the Herbarium of Tanta University’s Department of Pharmacognosy, Faculty of Pharmacy.

### 4.3. Extraction and Isolation of Different Compounds

*C. macrocarpa* roots were dried and powdered at room temperature, yielding 1 kg dry powder. They were extracted three times with 3 L of 95% methanol, each time for three days, and then concentrated using a rotary evaporator to provide a dry roots methanol extract (RME) of 7.25% yield. Total methanolic extract (4 g) was chromatographed on a silica gel column (160 g, ϕ 5 × 30 cm, fraction collected 75 mL) using the dry method and eluted successively with a gradient of CH_2_Cl_2_–MeOH mixtures of increasing polarities. The CH_2_Cl_2_–MeOH (97:3) eluate (A4, 530 mg) was subjected to CC using silica gel (20 g, ϕ 2 × 20 cm, fraction collected 15 mL) eluted successively with gradient CH_2_Cl_2_/MeOH. The CH_2_Cl_2_–MeOH (98:2) eluate (A4b, 130 mg) was subjected to Sephadex LH-20 to afford compound 1 (6 mg). The CH_2_Cl_2_–MeOH (94:6) eluate (A4d, 160 mg) was applied to isocratic CC with (95:5) CHCl_3_: MeOH to give a yellow residue, then purified on Sephadex LH-20 to give compound 2 (9 mg). The CH_2_Cl_2_–MeOH (90:10) eluate (A5, 327 mg) was re-chromatographed on silica gel (15 g, ϕ 1.5× 15 cm, fraction collected 10 mL) eluted in 2% increments with CHCl_3_–ethyl acetate to yield two subfractions. The third compound was obtained by purifying the second subfraction on Sephadex LH-20 with MeOH (7 mg).

### 4.4. LC-MS/MS Conditions

HPLC separation was accomplished using a (Waters) reversed-phase X select HSS T3 column (2.1×150 mm, 2.5 µm), a (Phenomenex) precolumn, and in-Line filter disks (0.5 µm × 3.0 mm). Adopting conditions described by [17]. PeakView^TM^ software was used to compare retention time and *m*/*z* values obtained by MS and MS^2^ to identify compounds. PeakView^TM^ software’s XIC Manager was used to calculate peak area values. For each targeted analyte, extracted ion chromatograms (XICs) were automatically generated and compared to a user-defined threshold [51]. The LC-MS/MS analysis was carried out in Proteomics and Metabolomics Unit, Children’s Cancer Hospital (57357).

### 4.5. Antibacterial Screening

Antibacterial screening was accomplished using the agar well diffusion method [52]. About 100 µL of suspensions of *S. enterica* isolates were spread on the Muller–Hilton agar plates’ surfaces. Three wells (with a diameter of 6 mm) were punched off by a cork borer. Each well was then filled with 100 μL (1024 µg/mL) of CRME, and the plates were incubated at 37 °C for 24 h using DMSO as a negative control and ciprofloxacin as a positive control.

#### 4.5.1. Determination of MICs

The MICs of CRME were detected for each tested isolate using the broth microdilution method, as previously described [53]. A positive control (bacteria without the extract) and negative control (broth without bacteria) were included in each microtitration plate. The lowest concentration of the extract that entirely inhibited the growth of bacteria (MIC) was determined by the absence of turbidity compared to the positive and negative controls. After determination of the MICs of CRME for each isolate, all the following tests were performed before and after treatment of the tested bacterial isolates with sub-inhibitory concentration (i.e., 0.5 MIC) of CRME.

#### 4.5.2. Integrity of Cell Membranes

The integrity of the bacterial cell membranes before and after treatment with the CRME was assessed by tracking the release of materials (DNA and RNA) which have an absorbance at 260 nm (A260), using a UV-Vis spectrophotometer (SHIMADZU, Kyoto, Japan) [54]. Bacterial cells were harvested after overnight growth using centrifugation and the produced pellets were resuspended in a solution of 0.5% NaCl. 

#### 4.5.3. Efflux Assay

This was performed using the fluorometric cartwheel method [55]. Tryptic soy agar with different concentrations of ethidium bromide (EtBr) (0.5, 1, 1.5, 2, 2.5 mg/L) was divided into parts by drawing radial lines. Using swabs, the bacterial cultures were inoculated onto the tryptic soy agar plates and incubated at 37 °C for 16 h. The plates were inspected by a UV-Vis transilluminator (SHIMADZU, Kyoto, Japan). The lowest concentration of EtBr that produced fluorescence by the isolates was detected. The tested isolates were grouped based on the measured EtBr minimum concentration into (a) Negative efflux activity for isolates that emitted fluorescence at 0.5 mg/L EtBr; (b) Intermediate efflux activity for isolates that emitted fluorescence at 1–2.0 mg/L EtBr; and (c) Positive efflux activity for isolates that emitted fluorescence at 2.5 mg/L EtBr.

#### 4.5.4. Antibiofilm Activity Assay

To evaluate the antibiofilm effect of CRME, a crystal violet assay was utilized, as previously described [56,57]. In brief, bacterial suspensions were prepared in tryptic soy broth (TSB) supplemented with 1% glucose in a 96-well microtitration plate. The plates were incubated aerobically at 37 °C for 24 h. The broth was then removed from each well and the wells were thoroughly washed with PBS, fixed using methyl alcohol, and stained with 1% *w*/*v* crystal violet. They were then washed and solubilized using 95% ethyl alcohol. The OD_570_ was detected using an ELISA reader (Sunrise Tecan, Männedorf, Austria). Both positive and negative growth controls were included in each plate. The antibiofilm activity of CRME was calculated as the percentage of reduction using the following equation:

$$\%\;\mathrm{Biofilm}\;\mathrm{reduction}=\frac{\mathrm{OD}\;\mathrm{growth}\;\mathrm{control}-\mathrm{OD}\;\mathrm{sample}\;}{\mathrm{OD}\;\mathrm{growth}\;\mathrm{control}}\times 100$$

#### 4.5.5. Impact on Biofilm Morphology by Light Microscope and SEM

Bacterial isolates, before and after treatment with CRME, were allowed to form biofilms on glass slides placed in the wells of 6-well plates. The biofilms were formed by flooding the glass slides with bacterial suspensions in Luria–Bertani (LB) broth and then incubated for 24 h. The LB broth was removed, and the formed biofilms were rinsed with 0.9% saline. The biofilms were then stained using crystal violet dye for 5 min. They were washed gently with distilled water and left to dry. The glass slides were then viewed using a light microscope (Labomed, Los Angeles, CA, US) [58].

In addition, SEM was utilized to view the formed bacterial biofilms on the surfaces of cover slides before and after treatment with CRME, as previously described [59,60]. After allowing the biofilms to be formed, they were fixed using 2% glutaraldehyde and 0.1 mol/L cacodylate buffer (pH 7.4) and then washed using 0.2 mol/L cacodylate buffer (pH 7.4). They were dehydrated by passing in: 30%, 50%, 70% and 90% ethyl alcohol followed by 100% ethyl alcohol two times. Finally, they were dried using a desiccator for 24 h, coated with gold-palladium, and viewed using SEM (Hitachi, Tokyo, Japan).

#### 4.5.6. qRT-PCR

The levels of expression of the genes encoding efflux pumps (*acr*A, *acr*B, *tol*C, and *oqx*B) [61] in addition to *spi*A and *agf*A genes which are associated with biofilm production [62] were measured using qRT-PCR, and16S rRNA gene was utilized as internal control according to Khosravani et al. [63]. After extraction of the total RNA from *S. enterica* isolates by Purelink^®^ RNA Mini Kit (Thermo SCIENTIFIC, Waltham, MA, USA), cDNA was synthesized using a power cDNA synthesis kit (iNtRON Biotechnology, Seoul, Korea) according to the manufacturer’s instructions. qRT-PCR was performed in the Rotor-Gene Q5 plex instrument (Qiagen, Hilden, Germany). Primers are listed in the Table S2. The 2^−ΔΔCt^ method was utilized to calculate the fold changes in the gene expression levels in *S. enterica* isolates after treatment, compared with those detected in their corresponding original non-treated isolates (their expression levels were set to be 1). According to Zheng et al. [64], only genes with ≥2-fold changes (increased or decreased) were statistically significant.

### 4.6. In Vivo Antidiarrheal Effect of C. macrocarpa Roots Methanol Extract

#### 4.6.1. Experimental Animals

In the current study, healthy Swiss albino mice (20–30 g) were purchased from the animal facility of Faculty of Pharmacy, Tanta, Egypt. They were housed in plastic cages for 2 weeks under standard conditions for adaptation of relative humidity and temperature. The experimental protocol was approved by the Research Ethical Committee of the Faculty of Pharmacy, Tanta University, Tanta, Egypt (FPTU-REC, PG-A-00105/2021). The management of the experimental mice was carried out as previously described [65].

#### 4.6.2. Experimental Design of the Antidiarrheal Study

Thirty male mice were distributed into five equal groups (*n* = 6). Group I (negative control group) mice were orally administered 10 mL/kg of the vehicle (20% DMSO). Group II (positive control group) mice were orally administered loperamide (3 mg/kg). Different doses of 100, 200 and 400 mg/kg of CRME were tested, orally, on groups III, IV and V of mice. Group II were administered the standard drug 15 min before the experiment. The administration of the negative control vehicle or the different doses of the crude extract was performed 30 min before the experiment.

#### 4.6.3. Diarrhea Induced with Castor Oil Oral Administration in Mice

This experiment was adopted according to a previously reported protocol [45]. Different groups (*n* = 6) of mice were abstained from food (but not water) for 18 h. Diarrhea was induced in mice by the oral receipt of 0.5 mL of castor oil, one hour after dosing (as previously mentioned. The mice were observed for 4 h, and each mouse was placed in a cage lined with a white paper which was changed every hour. The incidence of diarrhea, wet feces weight, total fecal content, and volume were determined. The percentages of fecal output and inhibition of defecation were determined by the equations:

(1) $$\%\;\mathrm{Inhibition}\;\mathrm{of}\;\mathrm{defecation}\;=\frac{\mathrm{Mean}\;\mathrm{fecal}\;\mathrm{weight}\;\mathrm{of}\;\mathrm{each}\;\mathrm{treatment}\;\mathrm{group}}{\mathrm{Mean}\;\mathrm{fecal}\;\mathrm{weight}\;\mathrm{of}\;\mathrm{control}\;\mathrm{group}}\times 100\;$$

(2) $$\%\;\mathrm{Inhibition}\;\mathrm{of}\;\mathrm{defecation}\;=\frac{\mathrm{Mo}-M}{\mathrm{Mo}}\times 100\;$$

Mo stands for the mean defecation of the control group; M stands for the mean defecation of the plant extract or the standard drug.

#### 4.6.4. Enteropooling Induction by Castor Oil Oral Administration in Mice

The purpose of this experiment was to evaluate intraluminal accumulation according to a literature procedure [46]. One hour following oral administration of the negative control vehicle, loperamide, 100, 200 and 400 mg/kg of CRME, each mouse was orally administered 0.5 mL of castor oil for induction of diarrhea. After 2 h, mice were killed by cervical dislocation, then the small intestine was separated and weighted. The intestinal contents were evacuated in a graduated tube and the intestine was weighed again. The difference between the full and empty intestines was determined. The percentage reduction in intestinal secretion and the intestinal contents weight were determined by the equations:

(3) $$\%\;\mathrm{Inhibition}\;\mathrm{of}\;\mathrm{using}\;\mathrm{MVSIC}\;=\frac{\mathrm{MVICC}-\mathrm{MVICT}}{\mathrm{MVICC}\;}\times 100\;$$

(4) $$\%\;\mathrm{Inhibition}\;\mathrm{of}\;\mathrm{using}\;\mathrm{MWSIC}\;=\frac{\mathrm{MWICC}\;-\;\mathrm{MWICT}}{\mathrm{MWICC}\;}\times 100\;$$

The MVSIC and MWSIC stand for the mean volume (V) or weight (W) of the small intestine contents. MVICC and MWICC stand for the mean volume (V) or weight (W) of the intestinal content for the group which administered the negative control. MVICT and MWICT stand for the mean volume (V) or weight (W) of the intestinal contents of the groups which administered the plant extract and the standard drug.

#### 4.6.5. Gastrointestinal Motility Test

The method of Carlo et al. [66] was adopted for testing gastrointestinal motility. the Swiss albino mice of each group were provided with castor oil (0.5 mL) to trigger diarrhea. Each mouse received 1 mL of 5% charcoal suspension in distilled water one hour after administration of each test dose. After that, mice were sacrificed by cervical dislocation, and the distance traveled by the charcoal suspension in the small intestine was measured (cm). The determination of the peristalsis index and percentage of inhibition was determined by the equation:

(5) $$\mathrm{Peristalsis}\;\mathrm{index}\;=\frac{\mathrm{Distance}\;\mathrm{traveled}\;\mathrm{by}\;\mathrm{charcoal}\;\mathrm{suspension}\times 100\;}{\mathrm{Length}\;\mathrm{of}\;\mathrm{the}\;\mathrm{small}\;\mathrm{intestine}\;}\;$$

(6) $$\%\;\mathrm{Inhibition}\;=\frac{\mathrm{Dc}\;-\;\mathrm{Dt}\times 100}{\mathrm{Dc}\;}$$

where Dc stands for the mean distance traveled in the group which administered the negative control and Dt stands for the mean distance traveled in the different tested groups.

### 4.7. Statistical Analysis

Genotypic patterns of ERIC-PCR were converted to numeric base pairs using the BioDocAnalyze program (Biometra, Germany). The data of ERIC-PCR were then transformed into a binary code (based on the bands’ presence or absence). Jaccard coefficient was utilized to determine the similarity of the profiles (Jaccard, 1912). The dendrogram was constructed via sequential hierarchical analysis and an unweighted pair group method with an arithmetic average (UPGMA). Both the dendrogram and the cluster analysis were accomplished using the SPSS software version 26 (IBM Corp., Rochester, MN, USA). All the tests were carried out in triplicate and the results were presented as mean ± SD (standard deviation). In general, the results were regarded to be significant if *p* < 0.05.

## 5. Conclusions

In the current investigation, the phytoconstituents of the CRME were identified using the LC- ESI-MS/MS technique for the first time. Altogether 39 compounds were identified, belonging to carboxylic acids, flavonoid, biflavonoid, polyflavonoid, catechins and stilbene derivatives. In addition, the phytochemical isolation of 2,3,2″,3″-tetrahydro-4′-*O*-methylamentoflavone, amentoflavone and 2,3-dihydrokaempferol-3-*O*-α-l-rhamnoside was achieved for the first time. The pure compounds, especially dihydrokaempferol-3-*O*-α-l-rhamnoside, exhibited a promising antibacterial activity with MIC value from 64 to 256 µg/mL. The current results showed that CRME presented a good activity on the tested *S. enterica* isolates. The antibacterial mechanism of CRME was deduced, as this plant could significantly reduce the bacterial membrane integrity in 45% of the treated isolates, the membrane depolarization of 40% of the isolates and the biofilm formation in eight isolates (40%). There was a significant increase, at *p* < 0.05, in the inner and outer membrane permeability, with percentages of 50% and 45%, respectively. *C. macrocarpa* could, remarkably, inhibit the efflux pump’s activity to restore sensitivity of the resistant bacteria towards different antibiotics in 55% of clinical isolates. *C. macrocarpa* exerted a significant downregulation effect on gene expression related to efflux activity and biofilm formation. The castor oil-induced diarrhea demonstrated that CRME showed a significant reduction (at 100, 200 and 400 mg/kg) in the defecation frequency in mice. In addition, it resulted in a significant reduction in the fluids accumulated in the small intestine at the dose of 400 mg/kg. CRME caused a significant delay in intestinal transit and suppressed gut motility at doses of 200 and 400 mg/kg with inhibition percentages of 64.95 and 80.24, respectively. The antidiarrheal index of CRME increased in a dose-dependent manner. Therefore, *C. macrocarpa* roots could be a future source for novel antimicrobial and antidiarrheal medications.

## Figures and Tables

**Table 1:** Metabolite Profiling of CRME identified by LC-MS/MS in negative mode.

No	Assignment	RT (min.)	(M–H)^−^ *m*/*z*	Formulas	Fragments or MS^2^ *m*/*z*
1	D-(-)-Quinic acid	1.234	191.056	C_7_H_12_O_6_	127.039, 171.030, 191.053
2	(-)-Shikimic acid	1.235	173.0456	C_7_H_10_O_5_	129.053, 137.021, 155.039, 173.043
3	Maleic acid	1.237	115.0018	C_4_H_4_O_4_	71.018, 114.999
4	Citraconic acid	1.299	129.0532	C_5_H_6_O_4_	29.050, 85.001
5	Procyanidin B2	4.592	577.1344	C_30_H_26_O_12_	289.021, 425.126, 577.134
6	Urocanic acid	4.745	137.0244	C_6_H_6_N2O_2_	137.024
7	(-)-Epicatechin	4.865	289.0729	C_15_H_14_O_6_	245.080, 289.072
8	Neohesperidin dihydrochalcone	5.273	611.1586	C_28_H_36_O_15_	543.161, 548.867, 611.165
9	Dihydrokaempferol-3-*O*-*α*-l-rhamnoside	5.355	433.1909	C_21_H_22_O_10_	179.059, 288.312, 342.957, 434.189
10	Apigenin-7-*O*-neohesperidoside (Rhoifolin)	5.677	577.1384	C_27_H_30_O_14_	269.043, 425.081, 532.910, 577.118
11	Procyanidin C1	5.677	865.1961	C_45_H_38_O_18_	289.201, 465.301, 865.194
12	Procyanidin B1	6.034	577.1349	C_30_H_26_O_12_	289.231, 425.102, 577.125
13	Naringenin-7-*O*-glucoside (Prunin)	6.209	433.1129	C_21_H_22_O_10_	271.065, 433.102, 433.196
14	Amentoflavone	6.221	537.194	C_30_H_18_O_10_	375.152, 399.210, 443.012, 537.189
15	*E*-3,4,5′-Trihydroxy-3′ glucopyranosylstilbene (Astringin)	6.482	405.1201	C_20_H_22_O_9_	243.067, 405.124
16	Isorhamnetin-3-*O*-rutinoside (Narcissin)	6.894	623.1995	C_28_H_32_O_16_	315. 211, 577.199, 623.171, 623.192
17	Luteolin-7-*O*-glucoside	6.982	447.0892	C_21_H_20_O_11_	285.035, 402.878, 447.084
18	Quercetin-3-D-xyloside	7.060	433.1666	C_20_H_18_O_11_	301.028, 326.927, 364.901, 433.164
19	5-Methoxysalicylic acid	7.061	167.0348	C_8_H_8_O_4_	152.010, 167.034
20	Isorhamnetin-3-*O*-glucoside	7.234	477.1055	C_22_H_22_O_12_	315.325, 454.144, 477.109
21	Ferulic acid	7.402	193.0874	C_10_H_10_O_4_	178.066, 193.085
22	Tetrahydro-4′-*O*-methyl amentoflavone	7.668	555.440	C_31_H_24_O_10_	541.022, 555.401
23	Apigenin-7-*O*-glucoside	7.805	431.0969	C_21_H_20_O_10_	269.042, 430.886, 431.103
24	Acacetin-7-*O*-rutinoside	7.855	591.1512	C_28_H_32_O_14_	283.0124, 392.898, 528.866, 591.144
25	Baicalein-7-*O*-glucuronide	7.994	445.1177	C_21_H_18_O_11_	112.987, 163.079, 269.353, 445.109
26	Kaempferol-3-glucuronide	8.006	461.1081	C_21_H_18_O_12_	285.331, 324.911, 392.897, 461.103
27	Quercetin	9.627	301.0349	C_15_H_10_O_7_	255.101, 301.031
28	Naringenin	10.183	271.0601	C_15_H_12_O_5_	93.040, 151.007, 271.057
29	Sinapyl aldehyde	11.077	207.0636	C_11_H_12_O_4_	192.0412, 207.065
30	3,3′,4′,5,7-pentahydroxyflavan	12.067	289.1812	C_15_H_14_O_6_	271.159, 289.180
31	3′-methoxy-4′,5,7-trihydroxyflavonol (isorhamnetin)	13.426	315.1947	C_16_H_12_O_7_	227.109, 283.168, 315.195
32	Hesperetin	16.591	301.1769	C_16_H_14_O_6_	301.178
33	Apigenin	16.725	269.1541	C_15_H_10_O_5_	269.157
34	Acacetin	18.035	283.1747	C_16_H_12_O_5_	268.153, 283.162
35	Esculin	18.257	339.1972	C_15_H_16_O_9_	295.205, 303.902, 339.191
36	Luteolin	18.330	285.1844	C_15_H_10_O_6_	269.158, 285.183
37	3,5,7-trihydroxy-4′-methoxyflavone (diosmetin)	20.352	299.2003	C_16_H_12_O_6_	231.101, 283.155, 299.199
38	Kaempferol-7-neohesperidoside	21.203	593.1549	C_27_H_30_O_15_	285.092, 389.172, 547.337, 593.131
39	Glycyrrhizate (glycyrrhizin)	26.874	821.3727	C_42_H_62_O_16_	685.426, 775.427, 821.3

**Table 2:** The change in efflux activity using the cartwheel method after treatment with CRME.

Before Treatment		After Treatment	
Minimum Conc. of EtBr (mg/L) (Number of Isolates)	Efflux Activity *	Minimum Conc. of EtBr (mg/L) (Number of Isolates)	Efflux Activity *
≤0.5 (3)	-(N)	≤0.5 (6)	-(N)
1 (1)	+(I)	1 (9)	+(I)
1.5 (1)	+(I)	1.5 (2)	+(I)
2 (2)	+(I)	2 (1)	+(I)
2.5 (13)	++(P)	2 (2)	++(P)

* Classification of efflux activity into (N) Negative efflux activity; (I) Intermediate efflux activity; and (P) Positive efflux activity.

**Table 3:** The relative expression of the genes encoding the efflux pump activity in *S. enterica* isolates after treatment with CRME.

Isolate Code	Relative Gene Expression *			
	*acr*A	*acr*B	*tol*C	*oqx*B
S1	**0.1 ± 0.3**	**0.2 ± 0.0**	1.1 ± 0.3	1.3 ± 0.2
S2	1.3 ± 0.1	**0.3 ± 0.1**	1.5 ± 0.3	1.0 ± 0.7
S3	1.4 ± 0.0	**0.2 ± 0.1**	1.7 ± 0.1	1.8 ± 0.2
S5	**0.1 ± 0.0**	1.1 ± 0.2	1.0 ± 0.4	1.1 ± 0.2
S6	1.3 ± 0.2	**0.2 ± 0.1**	1.2 ± 0.8	1.6 ± 0.2
S7	**0.5 ± 0.1**	1.0 ± 2.0	1.7 ± 0.2	1.5 ± 0.4
S8	**0.4 ± 0.0**	**0.1 ± 0.1**	1.4 ± 0.9	1.4 ± 0.3
S9	**0.2 ± 0.1**	**0.5 ± 0.1**	1.3 ± 0.0	0.9 ± 0.2
S15	0.9 ± 0.1	**0.2 ± 0.0**	1.2 ± 0.0	1.6 ± 0.2
S19	**0.4 ± 0.2**	**0.3 ± 0.2**	1.6 ± 0.7	1.7 ± 0.2
S20	**0.3 ± 0.0**	1.6 ± 0.2	1.3 ± 0.1	1.2 ± 0.0

* The bolded values refer to the decrease in the gene expression ≥ 2-fold.

**Table 4:** The relative expression (mean ± SD) of the tested genes related to biofilm formation in *S. enterica* isolates after treatment with CRME.

Isolate Code	Relative Gene Expression	Isolate Code
	*agf*A	*spi*A
S1	**0.4 ± 0.3**	**0.4 ± 0.2**
S2	**0.3 ± 0.1**	**0.1 ± 0.2**
S4	1.5 ± 0.3	**0.3 ± 0.2**
S6	**0.3 ± 0.2**	1.2 ± 0.1
S8	**0.4 ± 0.0**	**0.1 ± 0.1**
S9	0.9 ± 0.2	**0.2 ± 0.1**
S11	**0.2 ± 0.1**	1.8 ± 0.1
S13	**0.2 ± 0.0**	**0.1 ± 0.0**

**Table 5:** Antidiarrheal effect of CRME (results expressed as mean ± SD).

Treatment	The Onset of Diarrhea (min)	No. of Dry Feces	No. of Wet Feces	Weight of Dry Feces (g)	Weight of Wet Feces (g)	% Defecation Inhibition
Control	18.17 ± 1.5	11 ± 1.6	7.3 ± 1.2	1.8 ± 0.2	1.6 ± 0.0	-
Loperamide	100.33 ± 1.2	4 ± 0.8	1.7 ± 0.5	0.19 ± 0.0	0.1 ± 0.0	91.5
CRME (100 mg/kg)	68.5 ± 2.4	7 ± 0.8	3.7 ± 0.9	0.29 ± 0.0	0.19 ± 0.0	85.9
CRME (200 mg/kg)	87.6 ± 3.0	3.4 ± 0.5	2.4 ± 0.5	0.27 ± 0.0	0.14 ± 0.0	88
CRME (400 mg/kg)	108.5 ± 2.2	2.7 ± 0.5	1.7 ± 0.5	0.13 ± 0.0	0.09 ± 0.0	93.5

**Table 6:** Impact of CRME on the castor oil-induced enteropooling in mice.

Treatment	MVSIC * (mL)	% Inhibition in MVSIC	MWSIC ** (g)	% Inhibition in MWSIC
Control	0.71 ± 0.00	-	0.78 ± 0.00	-
Loperamide	0.23 ± 0.02	67.6	0.18 ± 0.01	77
CRME (100 mg/kg)	0.68 ± 0.00	4.23	0.71 ± 0.00	8.97
CRME (200 mg/kg)	0.61 ± 0.00	14.08	0.7±	10.26
CRME (400 mg/kg)	0.18±0.00	74.6	0.17±	78.2

* MVSIC: mean volume of the small intestinal content. ** MWSIC: mean weight of the small intestinal content. A significant reduction was caused by CRME in the MVSIC and MWSIC at the dose of 400 mg/kg b.w. compared to the control.

**Table 7:** Impact of CRME on the intestinal transit of charcoal in mice.

Treatment	Distance Traveled by Charcoal (cm)	Length of the Small Intestine (cm)	Peristalsis Index	% of Inhibition
Control	41 ± 0.07	50.2 ± 0.17	81.67	-
Loperamide	10.4 ± 0.22	51.3 ± 0.25	20.27	74.63
CRME (100 mg/kg)	37.37 ± 0.12	53.3 ± 0.21	70.11	8.85
CRME (200 mg/kg)	14.37 ± 0.26	52.4 ± 0.33	27.42	64.95
CRME (400 mg/kg)	8.1 ± 0.08	53.4 ± 0.22	15.16	80.24

**Table 8:** The in vivo antidiarrheal index of CRME.

Treatment	Delay in Defecation Time (Dfreq)	Gut Meal Travel Reduction (Gmeq)	Purging Frequency (Pfreq)	Antidiarrheal Index ADI
Control	-	-	-	-
Loperamide	452.17	74.63	91.5	145.62
CRME (100 mg/kg)	276.99	8.85	85.9	59.5
CRME (200 mg/kg)	382.11	64.95	88	129.74
CRME (400 mg/kg)	497.14	80.24	93.5	155.08

## Data Availability

The authors confirm that the data supporting this study are available within the article [and/or] its Supplementary Materials.

## Supplementary Materials

The following are available online, Figure S1: The total ion chromatogram (TIC) of metabolic profile of *C. macrocarpa* root extract Negative-mode, Table S1: MIC values of CRME against *S. enterica* isolates. Table S2: qRT–PCR primers. Figure S2. Structures of compounds tentatively identified in CRME by LC-MS/MS, Materials and Methods: Chemicals, Preparation of CRME for LC-MS/MS analysis, Bacterial isolates, ERIC-PCR, Inner and outer membrane permeability assays, Membrane depolarization assay, Acute oral toxicity of crude plant extract and assessment of in vivo antidiarrheal index [67,68,69].
