# Supplementary material for: Antidiarrheal and Antibacterial Activities of Monterey Cypress Phytochemicals: In Vivo and In Vitro Approach
Source: Molecules. 2022 Jan 6;27(2):346. doi: 10.3390/molecules27020346 (PMC8780600; doi:10.3390/molecules27020346)
Supplement: Supplementary file 1 [file molecules-27-00346-s001.zip › molecules-1513985-supplementary.pdf]

Supplementary data

Antidiarrheal and Antibacterial Activities of Monterey cypress phytochemicals: *In vivo* and *In vitro* Approach

Elshaymaa I. Elmongy [1,2,†], Walaa A. Negm [3,*,†], Engy Elekhnawy [4,*], Thanaa A. El-Masry [5], Nashwah G. M. Attallah [1,6], Najla Altwaijry [1], Gaber El-Saber Batiha [7], Suzy A. El-Sherbeni [3,*]

[1] Department of Pharmaceutical Sciences, College of Pharmacy, Princess Nourah bint Abdulrahman University, Riyadh 84428, Saudi Arabia; eielmongy@pnu.edu.sa (E.I.E.); ngmohamed@pnu.edu.sa (N.G.M.A.); naaltwaijry@pnu.edu.sa (N.A.)

[2] Pharmaceutical Chemistry Department, Faculty of Pharmacy, Helwan University, Helwan 11795, Egypt; Shaymaa.Taha@ pharm.helwan.edu.eg

[3] Pharmacognosy Department, Faculty of Pharmacy, Tanta University, Tanta 31111, Egypt; walaa.negm@pharm.tanta.edu.eg (W.A.N.); suzy.elsherbini@pharm.tanta.edu.eg (S.A.E.-S)

[4] Pharmaceutical Microbiology Department, Faculty of Pharmacy, Tanta University, Tanta 31111, Egypt; engy.ali@pharm.tanta.edu.eg (E.E.)

[5] Pharmacology Department, Faculty of Pharmacy, Tanta University, Tanta 31111, Egypt; thanaa.elmasri@pharm.tanta.edu.eg (T.A.E.-M)

[6] Egyptian Drug Authority (EDA), Giza 8655, Egypt (previously NODCAR)

[7] Department of Pharmacology and Therapeutics, Faculty of Veterinary Medicine, Damanhour University, Damanhour 22511, Egypt; gaberbatiha@gmail.com (G.E.B.)

† These authors contributed equally to this work.

* Correspondence: walaa.negm@pharm.tanta.edu.eg (W.A.N.); engy.ali@pharm.tanta.edu.eg (E.E.); suzy.elsherbini@pharm.tanta.edu.eg (S.A.E.-S)

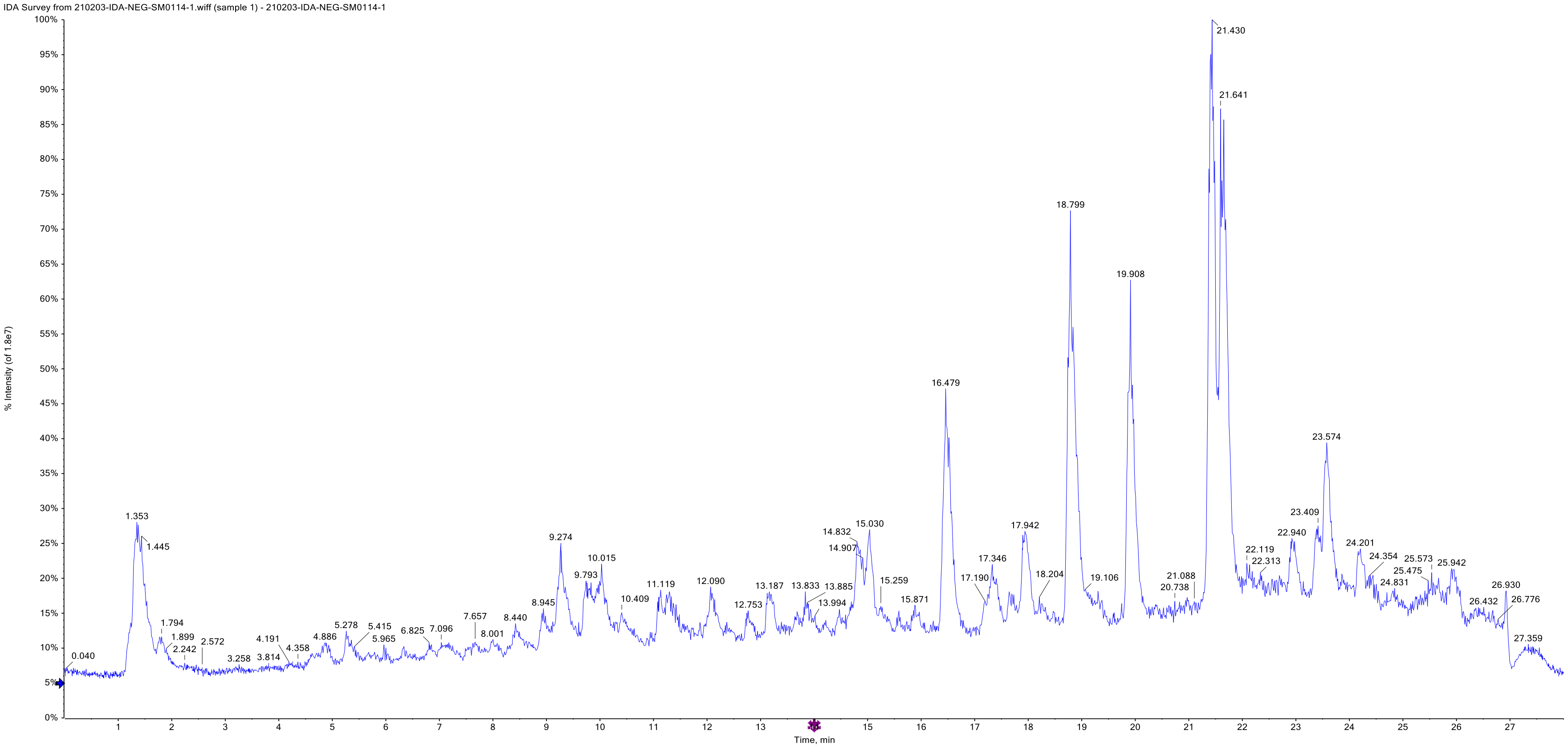

Figure S1: The total ion chromatogram (TIC) of metabolic profile of *C. macrocarpa* root methanol extract Negative-mode

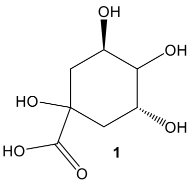
1

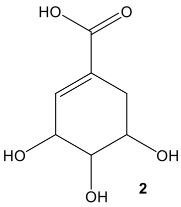
2

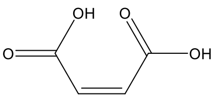
3

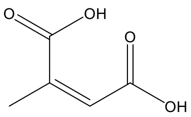
4

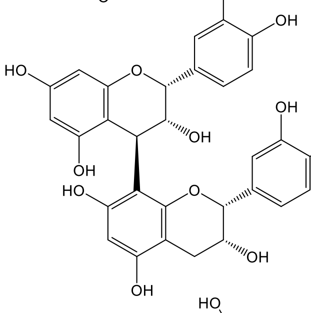
5

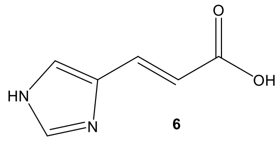
6

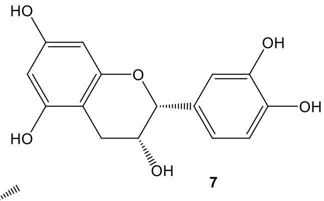
7

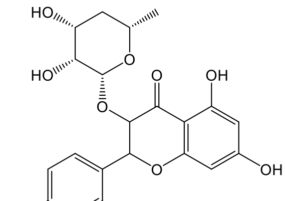
9

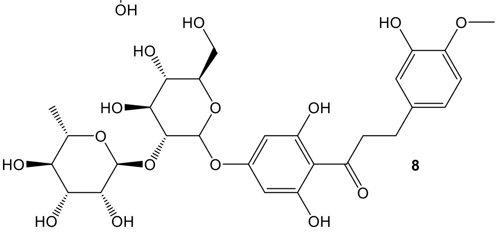
8

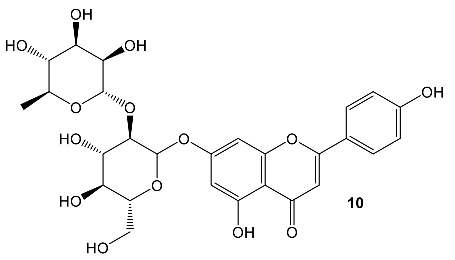
10

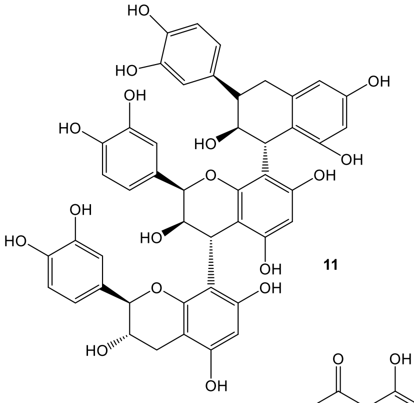
11

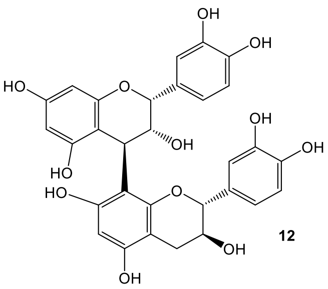
12

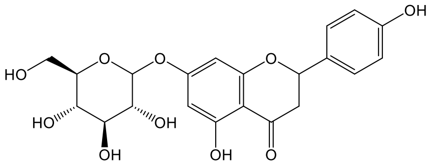
13

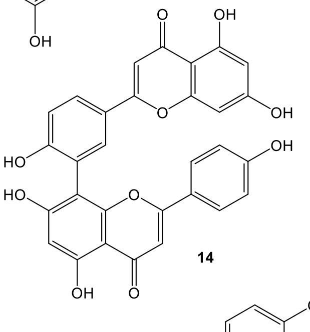
14

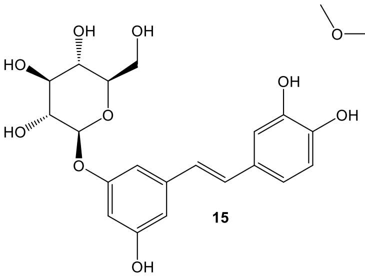
15

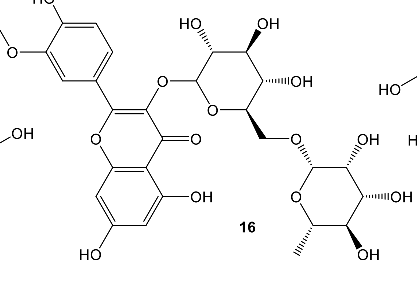
16

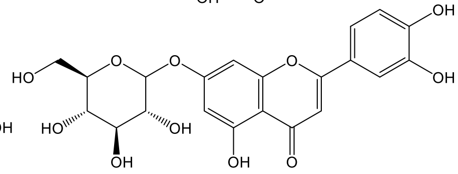
17

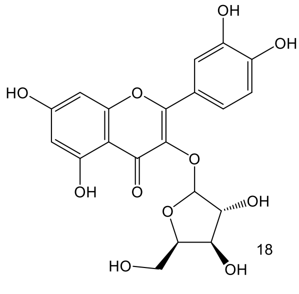
18

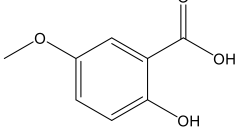
19

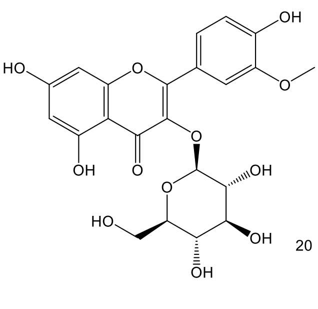
20

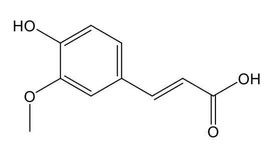
21

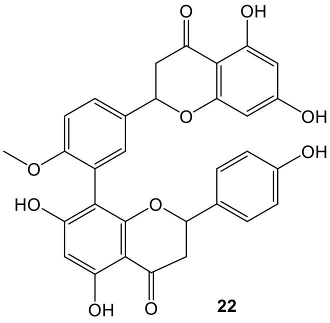

22

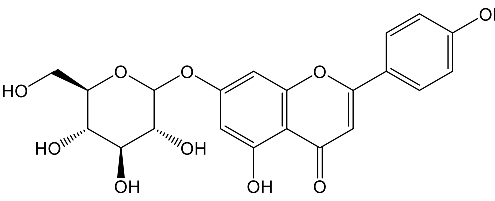

23

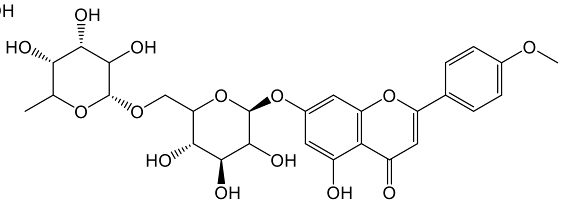

24

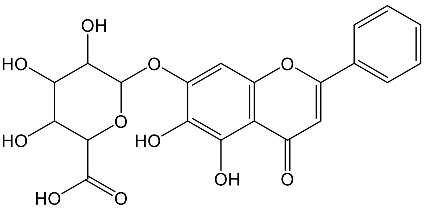

25

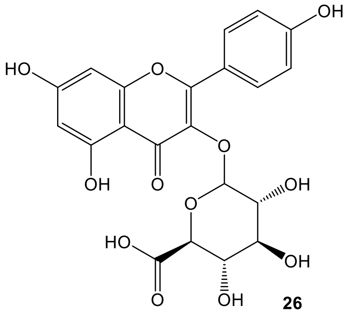

26

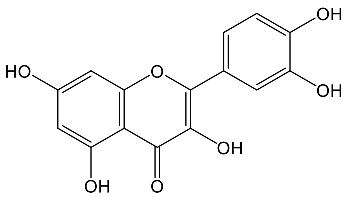

27

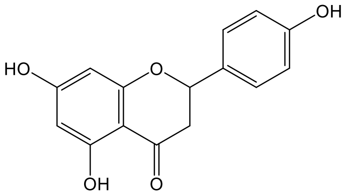

28

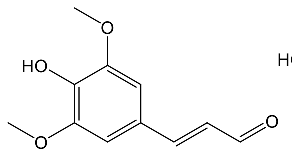

29

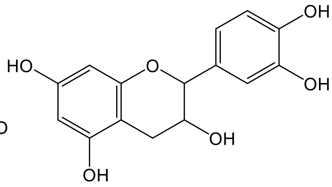

30

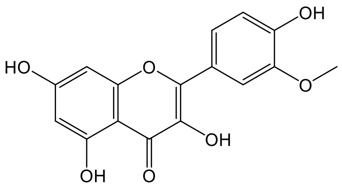

31

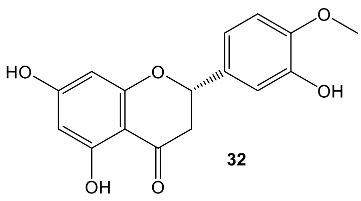

32

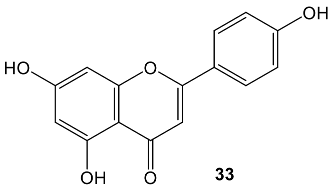

33

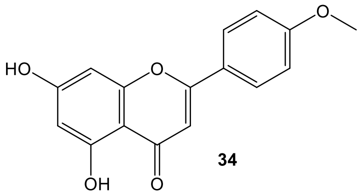

34

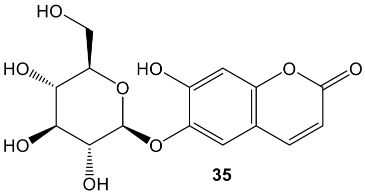

35

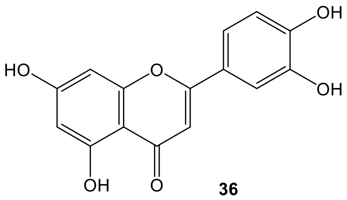

36

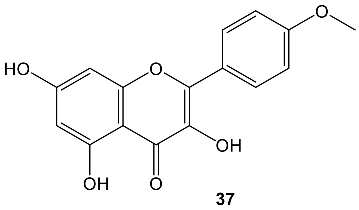

37

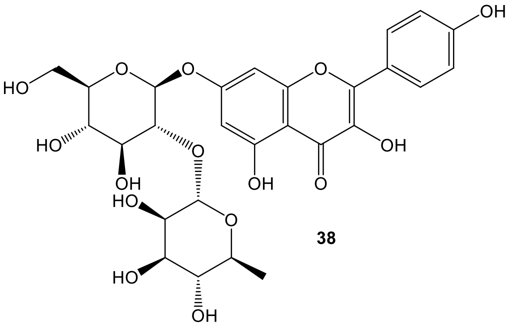

38

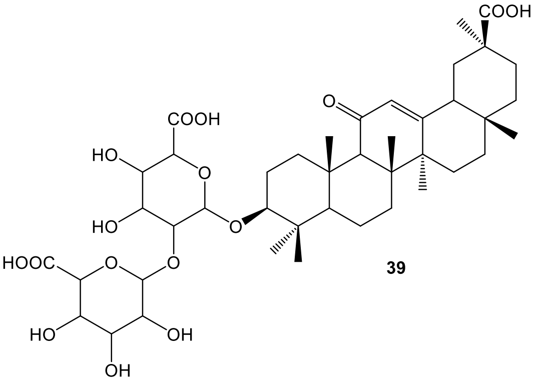

39

Figure. S2. Structures of compounds tentatively identified in CRME by LC-MS/MS.

Table S1. MIC values of CRME against *S. enterica* isolates.

Isolate	S1	S2	S3	S4	S5	S6	S7	S8	S9	S10
MIC value (μg/mL)	64	128	128	1024	128	64	256	256	256	64
**Isolate**	**S11**	**S12**	**S13**	**S14**	**S15**	**S16**	**S17**	**S18**	**S19**	**S20**
**MIC value (μg/mL)**	64	128	128	256	256	128	64	64	64	128

Table S2: qRT–PCR primers

**Tested genes**	**Primer**	**Sequence**
*acr*A	Forward	5'-TGTGGAACCAGTAATGCCGTC-3`
	Reverse	5'-GAAGCAGGAGCTGGCAAATG-3`
*acr*B	Forward	5'-GGCATTGGGTATGACTGGAC-3`
	Reverse	5'-GCATTACGGAGAACGGGATAG-3`
*tol*C	Forward	5'-TGCCGCAACTGGGTTTAGG-3`
	Reverse	5'-CAGAGAAGCGCTGGTTTCATTG-3`
*oqx*B	Forward	5'-TATCTCATTGGCGGCGTGAA-3`
	Reverse	5'-CGCGATTTTGGCGTTGATCT-3`
*agf*A	Forward	5'-TCCACAATGGGGCGGCGGCG-3`
	Reverse	5'-CCTGACGCACCATTACGCTG-3`
*spi*A	Forward	5'-CCAGGGGTCGTTAGTGTATTGCGTGAGATG-3`
	Reverse	5'-CGCGTAACAAAGAACCCGTAGTGATGGAT-3`
16S rRNA	Forward	5'-CGTGTTGTGAAATGTTGGGTTAA-3`
	Reverse	5'-CCGCTGGCAACAAAGGATAA-3`

Materials and Methods:

Chemicals

Acetonitrile (HPLC-grade) and ammonium formate were obtained from Sigma Aldrich (Germany). Sodium hydroxide and methanol (HPLC-grade) were purchased from Fisher Scientific (UK). Water (Milli-Q) was obtained from Millipore (USA). *bis*-(1,3-dibutyl barbituric acid) trimethineoxonol [DiBAC$_4$(3)] was purchased from Invitrogen. Sigma Aldrich provided all the analytical grade solvents used in this study.

Preparation of CRME sample for LC-MS/MS analysis

Two hundred grams of the powder of the dried root was macerated at room temperature in light petroleum ether to be defatted. After complete exhaustion, the powder was extracted with methanol, this extract was evaporated under vacuum at 40 °C. A weighed portion of the 50 mg dried residue was added to a 1 mL solution of (deionized water: methanol: acetonitrile (50: 25: 25). The plant sample in the previously mentioned solvent mixture was vortexed for 2 minutes, ultra-sonicated for 10 minutes, and centrifuged for another 10 minutes at 1000 g-force. Dilution with the reconstitution solvent and injection of 10 µL of the sample solution at a concentration of 1 µg/µL were carried out.

Bacterial isolates

Twenty *S. enterica* isolates were obtained from the culture collection at the pharmaceutical microbiology department, faculty of pharmacy, Tanta University. The clinical isolates were microscopically examined and were identified by different biochemical tests [67]. Then, suspected bacteria were cultured on Salmonella-Shigella (SS) agar, and they were further identified using API 20E test kit (bioMérieux, Inc., France), *S. enterica* (ATCC 35664) was used as a reference strain.

ERIC-PCR

Genotyping of isolates was carried out using ERIC-PCR. The sequence of the forward primer was (5′-ATG TAA GCT CCTGGG GAT TCA C-3′) and the reverse primer was (5′-AAG TAA GTG ACT GGGGTG AGC G-3′) [68]. After DNA extraction, the PCR reaction was carried out as previously described [68].

Inner membrane permeability assay

It was carried out by monitoring *β*-galactosidase enzyme release from the bacteria cytoplasm via measuring the enzyme activity on ONPG [55]. Briefly, after harvesting the bacterial isolates, they were resuspended in a solution of 0.5 % NaCl and 3.2 mL of the bacterial suspension was added to 300 µL of 34 mmol/ml ONPG solution. The produced ONP was detected over time via the determination of A420 by an ELISA reader (Sunrise Tecan, Austria).

Outer membrane permeability assay

This test was carried out before and after treatment as previously described [55]. The fluorescence of NBN solution (20 µmol/mL) was measured by a fluorescence spectrophotometer (SHIMADZU, Japan) at an excitation wavelength of 340 nm and an emission wavelength of 420 nm.

Membrane depolarization assay

It was performed using DiBAC4(3), which is a molecular probe, as previously described **[**56**]**. In brief, the bacterial suspensions, before and after treatment, were centrifuged and the pellets were resuspended in phosphate-buffered saline (PBS). The bacterial cells were then stained with 5 µg/mL DiBAC4(3). It is a fluorescent agent that can access the bacterial cells, when they are depolarized, and can bind to the intracellular membrane

proteins. When the cells are depolarized, they display an increase in the fluorescence measured using FACS verse flow cytometer (BD Biosciences, USA).

In vivo Antidiarrheal effect of C. macrocarpa roots methanol extract

Acute oral toxicity of crude plant extract

The OECD 425 guideline [69] was used for the detection of the acute oral toxicity of CRME in mice. They were subjected to fasting overnight, then they take their food after 4 hours of oral administration of different treatments. The mice were observed after administration of a single oral dose of 20% DMSO in saline (control) or 50, 100, 400, 1000, 2000, or 3000 mg/kg b.w. of the plant extract (each group n = 6). Each mouse was observed for any adverse reaction or mortality over the next 72 hours.

In vivo antidiarrheal index

The *in vivo* antidiarrheal index (ADI) was assessed by the equation:

$$\mathrm{ADI} = 3\sqrt{(\mathrm{D\ freq} \times \mathrm{G\ meq} \times \mathrm{P\ freq})} \quad (7)$$

D freq stands for the postponement in defecation time or diarrhea onset obtained from castor oil diarrheal test by:

$$\mathrm{D\ freq} = \frac{\text{mean onset of diarrhea in the test group} - \text{mean onset of diarrhea in the control} x\ 100}{\text{mean onset of diarrhea in the control group}} \quad (8)$$

G meq stands for the reduction of the gut meal travel obtained from the charcoal meal test (percentage of inhibition), and P freq stands for the purging rate obtained from the castor oil-induced diarrhea test (percentage of inhibition of defecation).
